# Comparative risk assessment of tobacco smoke constituents using the margin of exposure approach: the neglected contribution of nicotine

**DOI:** 10.1038/srep35577

**Published:** 2016-10-19

**Authors:** Claudia Baumung, Jürgen Rehm, Heike Franke, Dirk W. Lachenmeier

**Affiliations:** 1 Postgraduate Study for “Toxicology and Environmental Protection”, Institute for Legal Medicine, University of Leipzig, Leipzig, Germany; 2 Chemisches und Veterinäruntersuchungsamt (CVUA) Karlsruhe, Karlsruhe, Germany; 3 Epidemiological Research Unit, Technische Universität Dresden, Klinische Psychologie and Psychotherapie, Dresden, Germany; 4 Social and Epidemiological Research (SER) Department, Centre for Addiction and Mental Health (CAMH), Toronto, Canada; 5 Institute of Medical Sciences, University of Toronto (UofT), Toronto, Canada; 6 Dalla Lana School of Public Health, UofT, Toronto, Canada; 7 Dept. of Psychiatry, Faculty of Medicine, UofT, Toronto, Canada; 8 PAHO/WHO Collaborating Centre for Mental Health and Addiction, Toronto, Canada; 9 Rudolf Boehm Institut für Pharmakologie und Toxikologie, Medizinische Fakultät, Universität Leipzig, Leipzig, Germany

## Abstract

Nicotine was not included in previous efforts to identify the most important toxicants of tobacco smoke. A health risk assessment of nicotine for smokers of cigarettes was conducted using the margin of exposure (MOE) approach and results were compared to literature MOEs of various other tobacco toxicants. The MOE is defined as ratio between toxicological threshold (benchmark dose) and estimated human intake. Dose-response modelling of human and animal data was used to derive the benchmark dose. The MOE was calculated using probabilistic Monte Carlo simulations for daily cigarette smokers. Benchmark dose values ranged from 0.004 mg/kg bodyweight for symptoms of intoxication in children to 3 mg/kg bodyweight for mortality in animals; MOEs ranged from below 1 up to 7.6 indicating a considerable consumer risk. The dimension of the MOEs is similar to those of other tobacco toxicants with high concerns relating to adverse health effects such as acrolein or formaldehyde. Owing to the lack of toxicological data in particular relating to cancer, long term animal testing studies for nicotine are urgently necessary. There is immediate need of action concerning the risk of nicotine also with regard to electronic cigarettes and smokeless tobacco.

Tobacco smoking can cause numerous diseases such as cardiovascular disease, chronic obstructive pulmonary disease and various types of cancer including lung, oral, esophageal and bladder cancer^1^. Tobacco smoking has been classified as “carcinogenic to humans” (group 1) by the International Agency for Research on Cancer^2^. The World Health Organization (WHO) forecasts that cigarettes will kill nearly 10 million people per year globally by the year 2020^2^, and the reduction of smoking is crucial to achieve the goals of the “Global Action Plan for Prevention and Control of Non-Communicable Diseases (NCDs)”^3^,^4^.

Tobacco smoke is a complex chemical mixture containing more than 5000 components^2^,^5^,^6^,^7^,^8^,^9^,^10^. Classes of compounds include but are not limited to neutral gases, carbon and nitrogen oxides, amides, imides, lactames, carboxylic acids, lactones, esters, aldehydes, ketones, alcohols, phenols, amines, *N*-nitrosamines, *N*-heterocyclics, aliphatic hydrocarbons, monocyclic and polycyclic aromatic hydrocarbons, nitriles, anhydrides, carbohydrates, ethers, nitro compounds and metals^2^,^7^,^8^,^11^. The available knowledge on the relationship between tobacco smoking and the development of cancer in humans is based primarily on epidemiological evidence. A large amount of such evidence has become available over the last decades^2^. More than 70 carcinogens in tobacco smoke have been evaluated by the IARC monographs programme, and sixteen of these are classified as carcinogenic to humans into group 1^2^. Nicotine was not among the evaluated substances. Although nicotine is most commonly not considered as being carcinogenic itself, the risk of long-term nicotine intake is more or less unknown and under researched^12^. Some limited research shows that nicotine can stimulate the growth of lung cancer cells and may contribute to apoptosis^12^. Nevertheless, nicotine is currently not considered as a tobacco smoke constituent that is recommended by WHO for lowering^13^.

In the past, several studies provided lists of hazardous compounds in tobacco smoke. The various lists differ in composition of focused toxicants. The most established list is the so-called Hoffmann-list from the 1990s, which only includes carcinogens^5^. Most risk assessment studies are based on this Hoffmann list. This may be the reason why nicotine has been neglected.

There is significant evidence, that nicotine is the primary psychoactive component of tobacco smoke. Nicotine dependence, as defined by the International Classification of Diseases (ICD), is a chronic brain disorder, resulting from the interaction of several factors, and includes physical, psychological and social characteristics (for reviews see: refs 14, 15, 16).

Nicotine addictive effects are mediated through the binding to nicotinic acetylcholine receptor (nAChR) subtypes expressed in the brain, particularly located on dopaminergic neurons in the ventral tegmental area and stimulating the release of dopamine in the shell of the nucleus accumbens, which is the important mechanism in drug-induced reward (“brain’s reward system”)^17^,^18^,^19^. Additionally, changes in the dopamine level are supported by nicotine-induced release of other neurotransmitters, e.g. nicotine also augments glutamate release (which facilitates the release of dopamine) and γ-aminobutyric acid (GABA) release (which inhibits dopamine release) in these brain areas^14^,^17^,^18^.

The increase in dopamine level in the reward system signals a pleasurable experience. The repeated association with smoking-perceived positive effects in combination with a long-lasting conditioning to additional triggering stimuli promotes further nicotine consumption^16^,^20^. With long-term exposure to nicotine intake neuroadaptation (tolerance) develops, the existing nAChRs become less sensitive (desensitization/inactivation), which is accompanied by nicotine-induced upregulation of the receptor^18^,^21^.

Nicotine is rapidly and extensively (more than 80%^10^) metabolized by the liver, primarily by the liver enzyme CYP2A6 (and to a lesser extent by CYP2B6 and CYP2E1) to cotinine^22^. Variability in rate of metabolism contributes to vulnerability to tobacco dependence, response to smoking cessation treatment, and lung cancer risk^15^.

One of the currently most preferred approaches for quantitative risk assessment is the margin of exposure (MOE). The margin of exposure (MOE) is the ratio between a defined point on the dose-response curve for the adverse effect and the human intake. A suitable point of reference from the dose-response curve is the lower confidence limit of the dose which causes a benchmark response of 10% (BMDL10). The magnitude of the risk is reciprocally proportional to the MOE. In general for carcinogens an MOE of 10,000 or higher would be of low concern from a public health point of view and might be considered as a low priority for risk management. The more the MOE falls below 10,000, the higher is the risk of the substance for the considered toxicological endpoint.

Cunningham *et al*.^9^ presented a risk assessment of numerous tobacco smoke toxicants based on the MOE approach. For this, available toxicological data from the literature was used to calculate the BMDL10 and finally the MOE for each component with the objective to segregate the toxicants. A similar study from Xie *et al*.^23^ made a probabilistic risk assessment approach to prioritize the chemical constituents in mainstream smoke of cigarettes. For this purpose the MOE model was used as well. However both of the surveys did not include nicotine in the assessment.

Only a few previous studies researched the margin of exposure of nicotine but did not include the other tobacco toxicants. Lachenmeier and Rehm^24^ conducted a comparative risk assessment of drugs including alcohol and tobacco, the MOE of nicotine in tobacco being in the high risk category. Hahn *et al*.^25^ researched and assessed electronic cigarettes concerning chemical composition and exposure estimation based on the MOE approach. In this case, nicotine was the compound with the highest risk.

The intention of this study is therefore to provide a holistic comparative risk assessment of all tobacco and tobacco smoke components–for the first time including nicotine–using the MOE approach.

## Results

A summary of toxicological thresholds for various effects of nicotine is shown in Table 1. Literature data was used to determine the BMDL values for each effect (See data appendix provided as Supplementary Material for raw results of benchmark dose-response modelling). The values of BMDL10 depend on the researched toxic effect and range from 0.004 mg/kg bodyweight (bw) for various symptoms of intoxication (human (children)) and 0.2 mg/kg bw for toxic effects in the liver (rats) up to 3 mg/kg bw for mortality (various animal species, probabilistic calculation based on data from bird, dog, mouse and rat, see Lachenmeier and Rehm^24^ for details).

Based on the data from Cunningham *et al*.^9^, Xie *et al*.^23^ and own data (Lachenmeier and Rehm^24^), nicotine exposure from smoking cigarettes was calculated (Table 2).

As no information about the most likely function for intake distribution is available, a uniform probability distribution was entered into the calculation in this case (Supplementary Tables S1 and S2). The calculated mean values of daily nicotine intake range from 0.229 mg/kg bw/day up to 0.543 mg/kg bw/day.

**Figure 1:**
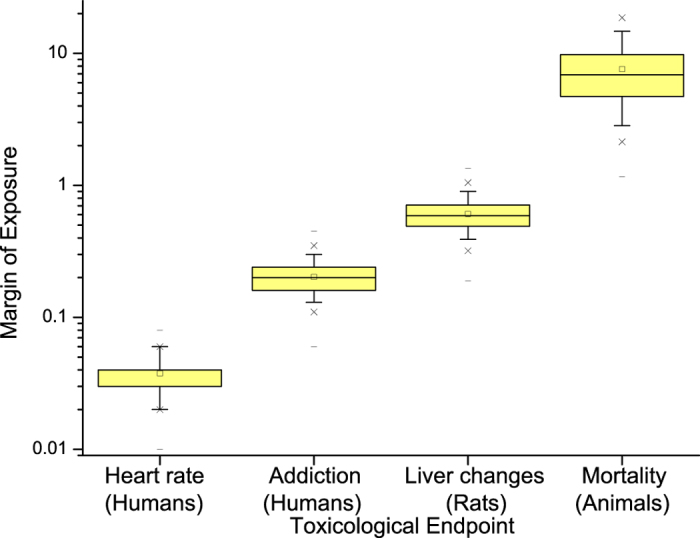
Margin of exposure for nicotine for daily smokers considering different toxicological endpoints (The box is determined by the 25th and 75th percentiles. The whiskers are determined by the 5th and 95th percentiles. 1st and 99th percentiles are marked by x, while minimum and maximum are marked with dash).

The margins of exposure for tobacco smoke constituents including nicotine are given in Table 3. The MOEs of the smoke constituents (except nicotine) are extracted from Xie *et al*.^23^ and Cunningham *et al*.^9^. For the toxicants that had several different MOEs tabulated for various endpoints, the lowest MOE was used in each case. Generally only toxicants with MOE below 10,000 were considered. The data for nicotine were calculated in this study based on the exposure data from Xie *et al*.^23^ and Cunningham *et al*.^9^, as well as our own data. The lowest MOE values of smoke toxicants based on the methodology of Xie *et al*.^23^ are in the range between 15 and 18 (hydrogen cyanide, 1,3-butadiene, acrolein). According to the methodology of Cunningham *et al*.^9^, the substances in tobacco smoke with the highest toxicological risks are acrolein, formaldehyde and cadmium compounds (mean MOE 1–8). Nicotine indicates even a higher potential toxicological risk; for four different toxicological endpoints and species, the MOEs of nicotine were calculated as ranging from 0.04 up to 7.6 (Fig. 1). The full numerical results of the MOE distributions are presented in Supplementary Table S3.

For sensitivity analysis, convergence testing during the probabilistic simulation was conducted. Convergence was achieved for all calculated output MOE values. This means that the generated output distributions are stable and reliable. The estimated means change less than 5% as additional iterations are run during the simulation. From the model input variables, the highest influence (as expressed by rank of regression coefficients) on the results is caused by the number of cigarettes per day, with the bodyweight on second position, and only a minor influence of nicotine yield per cigarette.

## Discussion

Some studies have performed a cumulative risk assessment on tobacco smoke toxicants by computing a total MOE. A total or cumulative MOE can be calculated by building the reciprocal of the sum of the reciprocals of the single MOEs. The implementation of this method was not possible in the current study because the toxicants have different target organs and effect mechanisms that makes it impossible to estimate a meaningful total MOE for tobacco toxicants.

This study allows comparing the potential toxicological risk of several tobacco smoke constituents especially in comparison with nicotine by using the MOE approach. For that purpose toxicity data of nicotine has been used to estimate the MOE and the results were compared to MOEs of various other tobacco toxicants from the literature. Due to the fact that adequate toxicity threshold data were unavailable for nicotine, BMDL values had to be calculated from literature dose-response data by own modelling. BMDL10 values of nicotine were derived for five toxicological endpoints (Table 1). For four of those endpoints the margins of exposure were calculated (Fig. 1). All four computed MOE distributions of nicotine are below 10 and may be interpreted as indicating a very high risk of this compound for tobacco smokers.

The MOE for the endpoint “heart rate acceleration” is based on data from Lindgren *et al*.^26^ who investigated the effect of nicotine in humans after i.v. dosage. As this endpoint is the most sensitive effect, the MOE is the lowest (0.04). However, this may be an overestimation of the risk. The detected heart rate increase of approximately 7 beats/min was still within the normal range of intraday fluctuations^27^ and is therefore not necessarily an adverse effect. The Lindgren *et al*.^26^ data is also statistically questionable for dose-response assessment, because the standard deviations of the data points were not provided and had to be estimated (see data appendix). In addition, a discrepancy exists in the study regarding the exposure units. On the one hand, the unit of nicotine is specified in the unit ng/ml plasma. However identical numerical values are mentioned in the results section as being in the unit μg/kg bw, which is not mathematically possible. The corresponding author was not able to clarify the discrepancy in the data (Lindgren 2015, personal communication). For these reasons, we believe that the MOE data based on Lindgren *et al*.^26^ cannot be judged as reliable and should be carefully scrutinized prior to their use as the basis for risk management action. It must be mentioned that EFSA^28^ judged Lindgren *et al*.^26^ as a *pivotal* study, which was exclusively used as the basis for EFSA’s risk assessment, the rationale probably being to provide the most conservative assessment.

The second considered effect was “addiction” in humans based on data from Benowitz and Henningfield^29^. The derived average MOE is 0.2. This is clearly in the high risk range and biologically plausible as nicotine is known as the addictive principle in tobacco^29^,^30^. Nevertheless, we are reluctant to use this value for risk assessment, as “addiction” is a rather vague concept which changed its definition remarkably often over the past 50 years, even if only medical classifications are considered^31^,^32^,^33^.

The other two endpoints rely on animal bioassays. For the effect “changes in rat liver” (fatty change, focal necrosis and dark cell change) an average MOE of about 0.61 was calculated based on data of Yuen *et al*.^10^. This calculation may, however, underestimate the risk of nicotine due to the very short duration of the liver toxicity study of only 10 days.

Finally, according to the severity of the toxicological endpoint, the average MOE of 7.6 for animal mortality is the highest (data based on several acute studies on various animal species summarized in Lachenmeier and Rehm^24^).

For the toxicological endpoint “various symptoms of intoxication in children” based on data from Woolf *et al*.^34^ the MOE was not calculated. The authors believe that this acute dermal exposure study in children is not applicable for risk assessment of habitual smoking, because of the different exposure conditions and questionable transferability to adults.

With a MOE from below 1 up to 7.6, the risk of nicotine is in the same dimension as the tobacco (smoke) toxicants with the lowest MOE such as acrolein, formaldehyde and cadmium compounds, which are the tobacco toxicants with the highest concerns relating to adverse health effects. There appears to be a fundamental problem that nicotine has never before been included in any risk assessment on tobacco or tobacco smoke toxicants. There are numerous studies about toxicological investigations from the tobacco industry and other authors available. Those studies tried to identify the most important toxicants from tobacco and to correlate them to the various diseases caused by cigarette smoking. However, nicotine has not been evaluated in any of them^5^,^6^,^13^,^35^,^36^. Up to now nicotine has not been associated with carcinogenesis but the risk of long-term nicotine intake is more or less unknown and under-researched^12^. The correlation of the disease risk to smoking dose, the mode of action and the etiology of the disease pathologies are not well understood^36^. Pankow *et al*.^37^ suggested that only about 4% of the observed risk for lung cancer can be explained by tobacco smoke toxicants. Also, in this case nicotine has not been part of the research. Could nicotine therefore explain a large part of the remaining risk? Currently there is only limited evidence to corroborate this hypothesis. Some experimental studies of different laboratories show that nicotine might promote or increase the risk of cancer^1^,^12^. West *et al*.^12^ demonstrated that it can stimulate the growth of lung cancer cells and may contribute to apoptosis. In alcohol, it had also been postulated for a long time, that ingredients other than ethanol were the main carcinogens, before ethanol itself was found as the main causal agent^38^,^39^. The correlation between smoking cigarettes and cancer is well established^2^. However, nicotine is so far considered as an addictive substance in tobacco smoke but not as a carcinogen. Bavarva *et al*.^40^ investigated the genomic influence of nicotine and its genotoxic mechanism mediated through oxidative stress in a cell line experiment. The results indicate that nicotine exposure can adversely affect the human genome by inducing somatic mutations and may contribute to increased cancer incidence, characterizing nicotine as a carcinogen or mutagen.

In addition to the ones located in the brain, nicotine receptors are found throughout the body; for example, in muscle, endothelia, kidney and skin, in normal lung and in lung tumors. These receptors are involved in a number of cellular pathways of carcinogenesis. That provides some mechanistic plausibility to the hypothesis that nicotine may contribute to the carcinogenic process^1^. But the studies have limitations in replicating human exposure and human evidence is clearly lacking so far. More research is needed to clarify if nicotine may make the major contribution to the mechanistically unexplained adverse effects. For example, there is no study on cancer endpoints currently available that would allow to calculate a MOE for this effect. We can only speculate if the pyrolysis products have been a misleading focus for research whereas the actual carcinogen has been neglected. Nicotine has previously not been evaluated by IARC but is on the current list of IARC to be re-evaluated with high priority^41^.

Our research showing that nicotine is among the top risk compounds in tobacco smoke leads to the question why there are no nicotine reduced cigarettes available on the market. Possibly the tobacco industry would lose clients if the nicotine exposures falls below the threshold of addiction^42^,^43^. It is proven that nicotine-reduced cigarettes would be less addictive^44^,^45^. For example, in a 10 week longitudinal study, the subjects at first smoked their usual brand followed by different types of research cigarettes with progressively lower nicotine content, each smoked for 1 week. After 4 weeks, 25% of the participants had spontaneously quit smoking^46^. The results of a 6-week research show that nicotine-reduced cigarettes lower its exposure, dependence and the number of cigarettes smoked^45^. These findings suggest that reducing the nicotine content of cigarettes could facilitate to quit smoking and therefore increase public health^45^. In the USA nicotine reduced cigarettes is a topic of major concern for research and policy since the commencement of an act in 2009^42^. Low-nicotine cigarettes have been and are on the US market^43^. However, they have not been successfully marketed owing to the issue of how much nicotine may be removed without affecting the taste. The tobacco industry did extensive research to determine the threshold of targeting nicotine dosing by cigarettes^42^. Benowitz and Henningfield^29^ derived a threshold level of nicotine in cigarettes from 5 mg or less to avert addiction. Instead Land *et al*.^47^ concluded that the tobacco manufacturers even increased the nicotine yield over the years.

In contrast, some studies showed that smoking nicotine reduced cigarettes leads to more intensive smoking to compensate for the necessity of nicotine^48^,^49^,^50^,^51^. For example, Ashton *et al*.^48^ suggested that smokers may compensate for about two-thirds of the difference in standard yields when switched from medium-nicotine to high or low-nicotine brands. The results show evidence of both upward and downward self-titration of nicotine intakes by smokers. This was confirmed by Gable^52^ who concluded that smokers tend to titrate to approximately 1 mg per cigarette by varying number and duration of puffs. Henningfield^49^ concluded that there are no health benefits on smoking cigarettes with lower (tar and) nicotine levels compared to “normal yields”. Woodward and Tunstall-Pedoe^51^ believe that in the case of deeper inhalation more carcinogenic pyrolysis products would be absorbed and concluded that not cigarettes with the lowest nicotine yields but those with low tar, low carbon monoxide and high nicotine yields appears to be the “most safe” cigarette. In contrast, Frenk and Dar^53^ do not confirm the “nicotine compensation hypothesis”. They are of the opinion that if nicotine is addictive in the same sense that heroin is, smokers would rapidly increase their dose (like heroin-dependent users do) by smoking more cigarettes or increasing number of puffs or deeper inhalation of the puffs or switching to cigarettes with a higher nicotine yield. But smokers do not. They arrive rapidly at their preferred number of cigarettes per day and the number remains stable for years. The conclusion of Frenk and Dar^53^ is that nicotine seems to be involved in a down-regulation mechanism but not in up-regulation. In fact light cigarettes may be smoked more intensely because of the sensory reward of their reduced tar and taste.

Another important aspect which needs to be considered is the fact that a high number of people use tobacco replacement or nicotine products over many years without getting cancer^54^, for instance pharmaceutical nicotine products for nicotine replacement therapy (NRT). NRT products such as nasal sprays, gums, tablets, lozenges and transdermal patches are marketed for helping people who want to stop smoking. The intended use of NRT products is the application for a fixed period of time. Only few users really quit smoking after this recommended time of 8–12 weeks. A lot of people do not stop the application after weeks but use these products over years some of them in addition to smoking. The US FDA does not have any significant safety concerns if smokers use nicotine replacement products in combination with another product or if they do not stop smoking completely before beginning to use smoking cessation products. NRTs do not appear to have a meaningful potential for abuse or dependence^55^. On the other hand, it may be difficult to monitor chronic risks such as cancer from over-the-counter products such as NRTs, and confounding to the co-consumption of NRT with smoked tobacco is difficult to exclude.

If we assume that nicotine alone or in the form of NRTs does not have any chronic effect, there could be more factors involved. One of those might be the type of nicotine exposure. The most precise difference in nicotine intake is the speed of effect. It takes only a few seconds for high doses of nicotine from a cigarette to reach the brain when inhaled. Medicinal products achieve lower levels over a period of minutes for products such as nasal spray or oral products and hours for transdermal patches^22^,^56^. Another essential point seems to be the complex mixture of nicotine and the other tobacco smoke toxicants. This is the exact difference from conventional cigarettes to electronic cigarettes (e-cigarettes) and NRTs. In conventional cigarettes, tobacco leaves are burned and nicotine is transferred besides toxic compounds produced during the combustion of tobacco such as carbon monoxide and nitrosamines from the solid phase to the aerosol. In e-cigarettes a nicotine solution is heated and nicotine is transferred from the liquid phase to the vapor phase. Because the harmful constituents of cigarette smoke are absent or significantly reduced in e-cigarette aerosols, smoking e-cigarettes is supposed to be less hazardous than smoking conventional cigarettes^57^,^58^. Hence, it may be speculated that nicotine and another compound in tobacco smoke may have cumulative effects.

In conclusion, all MOEs of nicotine in this study are less than 10 and within the range of very high risk. Owing to the lack of toxicological data particularly relating to cancer^30^, long term animal bioassay studies for nicotine are urgently necessary. In this context all kinds of possible nicotine exposures as well as certain co-exposures (tobacco smoke toxicants) and different nicotine concentrations related to a potential threshold of addiction need to be considered. The study from Lindgren *et al*.^26^ needs to be replicated. There is immediate need of action concerning the risk assessment of nicotine.

## Methods

Toxicity data on nicotine were obtained by a computer-assisted literature search. Searches were carried out in the following databases: PubMed, Toxnet and ChemIDplus (U.S. National Library of Medicine, Bethesda, MD), Web of Science (Thomson Scientific, Philadelphia, PA), and IPCS/INCHEM (International Programme on Chemical Safety/Chemical Safety Information from Intergovernmental Organizations, WHO, Geneva, Switzerland). We specifically aimed to identify clinical and epidemiological studies in humans and long-term animal studies that would be usable for dose-response modelling.

The methodology for quantitative risk assessment was based on a previous study for risk assessment of drugs^24^. The MOE approach was used for the risk assessment^59^,^60^. The MOE is defined as the ratio between the lower one-sided confidence limit of the BMD (BMDL) and estimated human intake of the same compound. If the BMDL as preferred toxicological threshold for MOE assessment is unavailable, no observed effect levels (NOEL), no observed adverse effect levels (NOAEL) or lowest observed adverse effect levels (LOAEL) may be applied. The values were either taken from the literature search, or additionally BMD and BMDL values were calculated using the US EPA’s BMDS 2.6 software (available at the US Environmental Protection Agency website: http://www.epa.gov/ncea/bmds/index.html). The human nicotine intake for smokers of cigarettes was based on literature data^9^,^23^,^24^.

The MOE was then calculated using the software package @Risk for Excel Version 5.5.0 (Palisade Corporation, Ithaca, NY, USA). Monte Carlo simulations were performed with 10,000 iterations using Latin Hypercube sampling and Mersenne Twister random number generator. Convergence was tested with a tolerance of 5% and a confidence level of 95%. The distribution functions and detailed calculation methodology is specified in Supplementary Tables S1 and S2.

## Additional Information

**How to cite this article**: Baumung, C. *et al*. Comparative risk assessment of tobacco smoke constituents using the margin of exposure approach: the neglected contribution of nicotine. *Sci. Rep.* **6**, 35577; doi: 10.1038/srep35577 (2016).

## Supplementary Material

Supplementary Information

## Figures and Tables

**Table 1:** Toxicological thresholds of nicotine.

Species	Effect	Type of endpoint	Value [mg/kg bw]	Reference
Humans, i.v., acute	Heart rate acceleration	LOAEL	0.008^a^	EFSA^28^ based on Lindgren *et al*.^26^
Humans, i.v., acute	Heart rate acceleration	BMDL for BMR = 1SD	0.013	Own modelling^c^ based on data from Lindgren *et al*.^26^
Humans, chronic cigarette use	Addiction	Threshold	0.07^b^	Benowitz and Henningfield^29^
Humans (Children), dermal, acute	Various symptoms of intoxication	LOEL	**0.01**	EFSA^28^ based on Woolf *et al*.^34^
Humans (Children), dermal, acute	Various symptoms of intoxication	BMDL10	0.004	Own modelling^c^ based on data from Woolf *et al*.^34^
Various animal species, acute	Mortality (LD50 studies)	BMDL10^d^	**3**	Lachenmeier and Rehm^24^
Rats, 10-day study	Liver: fatty change	BMDL10	0.27	Own modelling^c^ based on data from Yuen *et al*.^10^
Rats, 10-day study	Liver: focal necrosis	BMDL10	0.24	Own modelling^c^ based on data from Yuen *et al*.^10^
Rats, 10-day study	Liver: dark cell change	BMDL10	0.21	Own modelling^c^ based on data from Yuen *et al*.^10^
Rats, 10-day study	Pathological changes in liver	NOAEL	1.25	US EPA^61^ based on data from Yuen *et al*.^10^

^a^Based on a LOAEL of 0.0035 mg/kg bw using a correction factor of 0.44 (extrapolation from the intravenous route to the oral route). For methodology see EFSA^28^.

^b^Recalculated from the threshold level of 5 mg/day^29^ assuming an average bodyweight of 73.9 kg^62^.

^c^See data appendix provided as supplementary material for raw results of benchmark dose-response modelling.

^d^The BMDL10 was extrapolated from LD50 data in various animal studies in bird, dog, mouse and rat^24^.

**Table 2:** Data sources applied to calculate exposure for tobacco consumption-related toxicants and nicotine.

Study	Nicotine content per cigarette	Cigarette smoking per day	Bodyweight	Calculation method	Average daily nicotine intake (smokers)
Cunningham *et al*.^9^	Constituent yield from 1R4F cigarettes under the Health Canada Intense machine-smoke regime [note: the 1R4F reference cigarette contains 0.8 mg nicotine according to Calafat *et al*.^63^]	20 cigarettes/day	70 kg^64^	Point estimate	0.229 mg/kg bw/day^a^
Xie *et al*.^23^	Analyses of 30 brands of cigarettes sold in China using the Canadian intense smoking regime (2.09 ± 0.25 mg nicotine/cigarette)	Data from the 2006 China Health and Nutrition survey (average 16.4 cigarettes/day, P5 3 cigarettes/day, P95 30 cigarettes/day)	Average 63.3 kg, P5 46.7 kg, P95 84.2 kg	Probabilistic^b^	0.543 mg/kg bw/day^c^
Lachenmeier and Rehm^24^	1.65–1.89 mg nicotine/cigarette^47^	10–20 cigarettes/day^65^	73.9 ± 12 kg^62^	Probabilistic^b^	0.359 mg/kg bw/day

^a^Own calculation based on data from Cunningham *et al*.^9^.

^b^See Supplementary Tables S1 and S2 for distribution functions and calculation methodology.

^c^Own calculation based on data from Xie *et al*.^23^.

**Table 3:** Margin of exposure for tobacco smoke constituents summarized from the literature with comparison to own calculations for nicotine.

Study	Xie *et al*.^23^					Cunningham *et al*.^9^	
Constituent^a^	Point of departure (species/endpoint)	MOE Mean	MOE P5	MOE Median	MOE P95	Point of departure (species/endpoint)	MOE Mean
HCN	Human/Thyroid	15	4	9	46	not included in study	
1,3-Butadiene	Human/Leukaemia	18	4	10	52	Mice/Alveolar/bronchiolar adenoma and carcinoma	114
Acrolein	Rat/Nasal lesions	18	4	11	51	Rats/Laryngeal-epithelial squamous metaplasia	1
Acrylonitrile	Rat/Nasal histopathology	49	9	25	148	Rat/Flattening of respiratory epithelium nasal turbinates	42
Isoprene	Mouse/Spleen hemangiosarcoma	58	15	35	173	Mice/Nasal turbinate olfactory epithelial degeneration	325
Formaldehyde	Rat/Nasal cancer	102	21	59	307	Rats/Nasal squamous metaplasia	8
Acetaldehyde	Rat/Nasal tumours	166	42	100	490	Rats/Nasal adenocarcinoma	143
Cadmium compounds	Human/Kidney	196	23	65	499	Rats/Any lung tumours	6
Catechol	Rat/Glandular stomach hyperplasia	251	58	139	733	not included in study	
Benzene	Human/Decreased lymphocyte count	552	148	326	1596	not included in study	
Chromium	Rat/Respiratory	646	157	390	1941	not included in study	
Ammonia	Rat/Respiratory	1373	289	829	4201	not included in study	
Arsenic	Human/Lung cancer	1378	265	782	4283	not included in study	
Quinoline	Rat/Liver tumours	1528	373	920	4537	not included in study	
Pyridine	Mouse/Liver tumours	1552	391	936	4556	not included in study	
Styrene	Human/CNS effects	2644	645	1572	7765	not included in study	
NNK	Rat/Lung Cancer	3038	139	538	5857	Rats/Lung tumours	338
m/p-Cresol	Mouse/Nasal lesions	6735	1518	4023	20322	Mice/Lung bronchial hyperplasia	648
Ethylene oxide	not included in study					Mice/Alveolar/bronchiolar carcinoma or adenoma	2239
NNN	Rat/Nasal cavity tumours	263982	9554	37445	470255	Rats/Nasal tumours	3295
The following data for nicotine were calculated in this study based on the exposure data from Xie *et al*.^23^ and Cunningham *et al*.^9^							
Nicotine	Human/Heart rate acceleration (BMDL)	0.03	0.01	0.02	0.06	Human/Heart rate acceleration (BMDL)	0.06
Nicotine	Rats/Liver dark cell change	0.44	0.18	0.37	0.93	Rats/Liver dark cell change	0.92
Nicotine	Animals/Mortality	5	1	4	13	Animals/Mortality	11

The data from Xie *et al*.^23^ and Cunningham *et al*.^9^ are reprinted with permission from Elsevier.

^a^Only constituents with MOE below 10,000 were included from the literature studies. For Cunningham *et al*.^9^ the lowest MOE was selected for the constituents that had several different MOEs tabulated for various endpoints. The literature data were rounded to the nearest whole number in cases when decimals were provided.
