# Supplementary material for: Comparative risk assessment of tobacco smoke constituents using the margin of exposure approach: the neglected contribution of nicotine
Source: Sci Rep. 2016 Oct 19;6:35577. doi: 10.1038/srep35577 (PMC5069659; doi:10.1038/srep35577)
Supplement: Supplementary Information [file srep35577-s1.doc]

**Supplementary Tables S1-S3, Data appendix**

# Comparative risk assessment of tobacco smoke constituents using the margin of exposure approach: the neglected contribution of nicotine

Baumung, Claudia ^1,2^; Rehm, Jürgen ^3-8^; Franke, Heike ^1,9^; Lachenmeier, Dirk W. ^2,3*^

^1^Postgraduate Study for „Toxicology and Environmental Protection”, Institute for Legal Medicine, University of Leipzig, Leipzig, Germany,

^2^Chemisches und Veterinäruntersuchungsamt (CVUA) Karlsruhe, Karlsruhe, Germany,

^3^Epidemiological Research Unit, Technische Universität Dresden, Klinische Psychologie and Psychotherapie, Dresden, Germany,

^4^Social and Epidemiological Research (SER) Department, Centre for Addiction and Mental Health (CAMH), Toronto, Canada,

^5^Institute of Medical Sciences, University of Toronto (UofT), Toronto, Canada,

^6^Dalla Lana School of Public Health, UofT, Toronto, Canada,

^7^Dept. of Psychiatry, Faculty of Medicine, UofT, Toronto, Canada,

^8^PAHO/WHO Collaborating Centre for Mental Health and Addiction, Toronto, Canada,

^9^Rudolf Boehm Institut für Pharmakologie und Toxikologie, Medizinische Fakultät, Universität Leipzig, Leipzig, Germany

* Correspondence and requests for materials should be addressed to

D.W.L. (Lachenmeier@web.de)

Supplementary Table S1. Distribution functions as input for probabilistic analysis

| **Parameter** | **Risk function ^a^** | **Unit** |
| --- | --- | --- |
| Bodyweight for analysis according to Lachenmeier and Rehm (2015) | RiskNormal(73.9;12) | kg |
| Bodyweight for analysis in comparison to Xie *et al.* (2012) | RiskNormalAlt("mu";63.3;95%;84.2) | kg |
| Bodyweight for analysis in comparison to Cunningham et al. (2011) | 70 ^b^ | kg |
| Cigarettes per day for analysis according to Lachenmeier and Rehm (2015) | RiskUniform(10;20) | - |
| Cigarettes per day for analysis in comparison to Xie *et al.* (2012) | RiskNormalAlt("mu";16.4;95%;30;RiskTruncate(5;40)) | - |
| Cigarettes per day for analysis in comparison to Cunningham *et al.* (2011) | 20 ^b^ | - |
| Nicotine yield per cigarette for analysis according to Lachenmeier and Rehm (2015) | RiskUniform(1.65;1.89) | mg |
| Nicotine yield per cigarette for analysis in comparison to Xie et al. (2012) | RiskBetaGeneral(1.2144;1.4006;1.64582;2.61184;RiskTruncate(0;)) | mg |
| Nicotine yield per cigarette for analysis in comparison to Cunningham *et al.* (2011) | 0.8 ^b^ | mg |
| BMDL(Human heart rate) | 0.013 ^b^ | mg/kg bw/day |
| BMDL(Human addiction) | 0.07 ^b^ | mg/kg bw/day |
| BMDL(Rat Liver changes) | 0.21 ^b^ | mg/kg bw/day |
| BMDL(Animal mortality) according to Lachenmeier and Rehm (2015) | RiskNormal(2;1.8;RiskTruncate(0.92;5) | mg/kg bw/day |

^a^ RiskNormal(mean;standard deviation) specifies a normal distribution with the entered mean and standard deviation. RiskNormalAlt(arg1type, arg1value, arg2type,arg2value) specifies a normal distribution with two arguments of the type arg1type and arg2type. These arguments can be either a percentile between 0 and 1 or mu or sigma. RiskUniform(minimum;maximum) specifies a uniform probability distribution with the entered minimum and maximum values. Every value across the range has an equal likelihood of occurrence (“no knowledge” distribution). RiskTruncate(minimum;maximum) truncates the input distribution. Truncating distribution restricts samples drawn from the distribution to values within the entered minimum-maximum range. RiskBetaGeneral(alpha1,alpha2,minimum,maximum) specifies a beta distribution with the defined minimum and maximum using the shape parameters alpha1 and alpha2.

^b^ calculation as point estimate (no distribution available)

**Supplementary Table S2. Detailed calculation methodology for probabilistic risk assessment of nicotine due to tobacco smoking**

| **Parameter** | **Calculation formula** **for the software package @Risk for Excel Version 5.5.0 (Palisade, Corporation, Ithaca, NY, USA) ^a^** | **Unit** |
| --- | --- | --- |
| Nicotine intake per day | = Risk function for cigarettes per day * Risk function for Nicotine yield per cigarette | mg/day |
| Nicotine intake per kg bodyweight per day | = Nicotine intake per day / Risk function for bodyweight | mg/kg bw/day |
| MOE | = Risk function for BMDL / Nicotine intake per kg bodyweight per day | - |

**Supplementary Table S3. Raw results of probabilistic estimation of nicotine intakes and margin of exposure (MOE) using 10,000 iterations**

| **Parameter / Reference for calculation method** | **Minimum** | **Maximum** | **Mean** | **Std Deviation** | **5% Perc** | **25% Perc** | **50% Perc** | **75% Perc** | **90% Perc** | **95% Perc** |
| --- | --- | --- | --- | --- | --- | --- | --- | --- | --- | --- |
| Nicotine intake mg per day (Lachenmeier and Rehm 2015) | 17 | 38 | 27 | 5 | 19 | 22 | 26 | 31 | 34 | 35 |
| *Nicotine intake mg per day (Xie et al. 2012)* | *8* | *99* | *37* | *16* | *15* | *25* | *36* | *47* | *58* | *65* |
| Nicotine intake mg per day (Cunningham *et al.* 2011) | - | - | 16 | - | - | - | - | - | - | - |
| Nicotine intake mg per kg bodyweight per day (Lachenmeier and Rehm 2015) | 0.16 | 1.10 | 0.37 | 0.10 | 0.23 | 0.30 | 0.36 | 0.43 | 0.50 | 0.54 |
| Nicotine intake mg per kg bodyweight per day (Xie *et al.* 2012) | 0.11 | 15.87 | 0.61 | 0.33 | 0.23 | 0.40 | 0.57 | 0.77 | 1.00 | 1.15 |
| Nicotine intake mg per kg bodyweight per day (Cunningham *et al.* 2011) | - | - | 0.23 | - | - | - | - | - | - | - |
| MOE for human heart rate (Lachenmeier and Rehm 2015) | 0.01 | 0.08 | 0.04 | 0.01 | 0.02 | 0.03 | 0.04 | 0.04 | 0.05 | 0.06 |
| MOE for human heart rate (Xie *et al.* 2012) | 0.00 | 0.12 | 0.03 | 0.02 | 0.01 | 0.02 | 0.02 | 0.03 | 0.05 | 0.06 |
| MOE for human heart rate (Cunningham *et al.* 2011) | - | - | 0.06 | - | - | - | - | - | - | - |
| MOE for human addiction (Lachenmeier and Rehm 2015) | 0.06 | 0.45 | 0.20 | 0.05 | 0.13 | 0.16 | 0.20 | 0.24 | 0.28 | 0.30 |
| MOE for human addiction (Xie *et al.* 2012) | 0.00 | 0.65 | 0.15 | 0.08 | 0.06 | 0.09 | 0.12 | 0.18 | 0.26 | 0.31 |
| MOE for human addiction (Cunningham *et al.* 2011) | - | - | 0.31 | - | - | - | - | - | - | - |
| MOE for rat Liver changes (Lachenmeier and Rehm 2015) | 0.19 | 1.35 | 0.61 | 0.16 | 0.39 | 0.49 | 0.59 | 0.71 | 0.83 | 0.90 |
| MOE for rat Liver changes (Xie *et al.* 2012) | 0.01 | 1.95 | 0.44 | 0.24 | 0.18 | 0.27 | 0.37 | 0.53 | 0.77 | 0.93 |
| MOE for rat Liver changes (Cunningham *et al.* 2011) | - | - | 0.92 | - | - | - | - | - | - | - |
| MOE for animal mortality (Lachenmeier and Rehm 2015) | 1.17 | 27.86 | 7.60 | 3.76 | 2.83 | 4.71 | 6.89 | 9.76 | 12.78 | 14.72 |
| MOE for animal mortality (Xie *et al.* 2012) | 0.15 | 41.13 | 5.48 | 4.02 | 1.46 | 2.79 | 4.40 | 6.83 | 10.43 | 13.24 |
| MOE for animal mortality (Cunningham *et al.* 2011) | 4.03 | 21.87 | 11.47 | 4.60 | 4.80 | 7.63 | 11.02 | 14.90 | 18.18 | 19.73 |

Data appendix with raw result for benchmark dose-response modelling

1. BMD Modelling for heart rate acceleration with data from Lindgren at al. (1999): raw data from Figure 1, right panel. Maximum beats/min values assumed to be 63/66/67/73/78 for the dose groups 0/3.5/7/14/28 µg/kg bw. Standard deviation not provided and estimated as 10 beats/min according to Moser *et al.* (1994) Circulation 90:1078-82.

====================================================================

Power Model. (Version: 2.18; Date: 05/19/2014)

Input Data File: C:/USEPA/BMDS2601/Data/pow_Lindgren_Lindren.(d)

Gnuplot Plotting File: C:/USEPA/BMDS2601/Data/pow_Lindgren_Lindren.plt

Thu Aug 20 16:52:31 2015

====================================================================

BMDS Model Run

~~~~~~~~~~~~~~~~~~~~~~~~~~~~~~~~~~~~~~~~~~~~~~~~~~~~~~~~~~~~~~~~~~~~~

The form of the response function is:

Y[dose] = control + slope * dose^power

Dependent variable = Max_beats_per_min

Independent variable = Dose

rho is set to 0

The power is restricted to be greater than or equal to 1

A constant variance model is fit

Total number of dose groups = 5

Total number of records with missing values = 0

Maximum number of iterations = 500

Relative Function Convergence has been set to: 1e-008

Parameter Convergence has been set to: 1e-008

Default Initial Parameter Values

alpha = 100

rho = 0 Specified

control = 63

slope = 290.43

power = -9999

Asymptotic Correlation Matrix of Parameter Estimates

( *** The model parameter(s) -rho -power

have been estimated at a boundary point, or have been specified by the user,

and do not appear in the correlation matrix )

alpha control slope

alpha 1 -8.1e-009 -8.1e-009

control -8.1e-009 1 -0.73

slope -8.1e-009 -0.73 1

Parameter Estimates

95.0% Wald Confidence Interval

Variable Estimate Std. Err. Lower Conf. Limit Upper Conf. Limit

alpha 93.7721 15.8504 62.706 124.838

control 63.775 1.6872 60.4681 67.0819

slope 535.714 116.916 306.563 764.866

power 1 NA

NA - Indicates that this parameter has hit a bound

implied by some inequality constraint and thus

has no standard error.

Table of Data and Estimated Values of Interest

Dose N Obs Mean Est Mean Obs Std Dev Est Std Dev Scaled Res.

------ --- -------- -------- ----------- ----------- ----------

0 14 63 63.8 10 9.68 -0.299

0.0035 14 66 65.6 10 9.68 0.135

0.007 14 67 67.5 10 9.68 -0.203

0.014 14 73 71.3 10 9.68 0.667

0.028 14 78 78.8 10 9.68 -0.299

Model Descriptions for likelihoods calculated

Model A1: Yij = Mu(i) + e(ij)

Var{e(ij)} = Sigma^2

Model A2: Yij = Mu(i) + e(ij)

Var{e(ij)} = Sigma(i)^2

Model A3: Yij = Mu(i) + e(ij)

Var{e(ij)} = Sigma^2

Model A3 uses any fixed variance parameters that

were specified by the user

Model R: Yi = Mu + e(i)

Var{e(i)} = Sigma^2

Likelihoods of Interest

Model Log(likelihood) # Param's AIC

A1 -193.587177 6 399.174355

A2 -193.587177 10 407.174355

A3 -193.587177 6 399.174355

fitted -193.930374 3 393.860748

R -203.111216 2 410.222432

Explanation of Tests

Test 1: Do responses and/or variances differ among Dose levels?

(A2 vs. R)

Test 2: Are Variances Homogeneous? (A1 vs A2)

Test 3: Are variances adequately modeled? (A2 vs. A3)

Test 4: Does the Model for the Mean Fit? (A3 vs. fitted)

(Note: When rho=0 the results of Test 3 and Test 2 will be the same.)

Tests of Interest

Test -2*log(Likelihood Ratio) Test df p-value

Test 1 19.0481 8 0.0146

Test 2 5.68434e-014 4 1

Test 3 5.68434e-014 4 1

Test 4 0.686393 3 0.8764

The p-value for Test 1 is less than .05. There appears to be a

difference between response and/or variances among the dose levels

It seems appropriate to model the data

The p-value for Test 2 is greater than .1. A homogeneous variance

model appears to be appropriate here

The p-value for Test 3 is greater than .1. The modeled variance appears

to be appropriate here

The p-value for Test 4 is greater than .1. The model chosen seems

to adequately describe the data

Benchmark Dose Computation

Specified effect = 1

Risk Type = Estimated standard deviations from the control mean

Confidence level = 0.95

BMD = 0.0180761

BMDL = 0.0130478

2. BMD-Modelling with data from Woolf *et al.* (1997): raw data from Table 4. Upper bound set as dose level (i.e. 0.01, 0.099 and 0.5 mg/kg bw).

====================================================================

Multistage Model. (Version: 3.4; Date: 05/02/2014)

Input Data File: C:/USEPA/BMDS2601/Data/msc_Woolf_Woolf.(d)

Gnuplot Plotting File: C:/USEPA/BMDS2601/Data/msc_Woolf_Woolf.plt

Thu Aug 20 15:38:26 2015

====================================================================

BMDS_Model_Run

~~~~~~~~~~~~~~~~~~~~~~~~~~~~~~~~~~~~~~~~~~~~~~~~~~~~~~~~~~~~~~~~~~~~~

The form of the probability function is:

P[response] = background + (1-background)*[1-EXP(

-beta1*dose^1-beta2*dose^2)]

The parameter betas are restricted to be positive

Dependent variable = Perc

Independent variable = Dose

Total number of observations = 3

Total number of records with missing values = 0

Total number of parameters in model = 3

Total number of specified parameters = 0

Degree of polynomial = 2

Maximum number of iterations = 500

Relative Function Convergence has been set to: 1e-008

Parameter Convergence has been set to: 1e-008

Default Initial Parameter Values

Background = 0

Beta(1) = 0

Beta(2) = 4.07602e+020

Asymptotic Correlation Matrix of Parameter Estimates

( *** The model parameter(s) -Beta(1)

have been estimated at a boundary point, or have been specified by the user,

and do not appear in the correlation matrix )

Background Beta(2)

Background 1 -0.33

Beta(2) -0.33 1

Parameter Estimates

95.0% Wald Confidence Interval

Variable Estimate Std. Err. Lower Conf. Limit Upper Conf. Limit

Background 0.287085 0.172829 -0.0516538 0.625824

Beta(1) 0 NA

Beta(2) 36.5969 73.4008 -107.266 180.46

NA - Indicates that this parameter has hit a bound

implied by some inequality constraint and thus

has no standard error.

Analysis of Deviance Table

Model Log(likelihood) # Param's Deviance Test d.f. P-value

Full model -5.60136 3

Fitted model -5.60168 2 0.000640384 1 0.9798

Reduced model -8.96765 1 6.73259 2 0.03452

AIC: 15.2034

Goodness of Fit

Scaled

Dose Est._Prob. Expected Observed Size Residual

------------------------------------------------------------------------

0.5000 0.9999 4.000 4.000 4.000 0.017

0.0990 0.5020 1.004 1.000 2.000 -0.006

0.0100 0.2897 2.028 2.030 7.000 0.002

Chi^2 = 0.00 d.f. = 1 P-value = 0.9853

Benchmark Dose Computation

Specified effect = 0.1

Risk Type = Extra risk

Confidence level = 0.95

BMD = 0.0536558

BMDL = 0.00399136

BMDU = 0.173017

Taken together, (0.00399136, 0.173017) is a 90 % two-sided confidence

interval for the BMD

Cancer Slope Factor = 25.0541

3. BMD-modelling for Yuen *et al.* (1995) study. Data from table 1 (non-pregnant rats). The sum of all degrees of changes (i.e. mild, moderate and sever) in liver morphology were used as input variables. The endpoint confluent necrosis was not modelled due to lack in dose-response (only one positive dose group).

Endpoint 1: Fatty change

====================================================================

Logistic Model. (Version: 2.14; Date: 2/28/2013)

Input Data File: C:/USEPA/BMDS250/Data/lnl_Nicotine Yuen et al. 1995_Nicotine Yuen et al. 1995.(d)

Gnuplot Plotting File: C:/USEPA/BMDS250/Data/lnl_Nicotine Yuen et al. 1995_Nicotine Yuen et al. 1995.plt

Thu Feb 05 09:51:37 2015

====================================================================

BMDS_Model_Run

~~~~~~~~~~~~~~~~~~~~~~~~~~~~~~~~~~~~~~~~~~~~~~~~~~~~~~~~~~~~~~~~~~~~~

The form of the probability function is:

P[response] = background+(1-background)/[1+EXP(-intercept-slope*Log(dose))]

Dependent variable = FattyChange

Independent variable = Dose

Slope parameter is restricted as slope >= 1

Total number of observations = 3

Total number of records with missing values = 0

Maximum number of iterations = 500

Relative Function Convergence has been set to: 1e-008

Parameter Convergence has been set to: 1e-008

User has chosen the log transformed model

Default Initial Parameter Values

background = 0.333333

intercept = -1.03625

slope = 4.64386

Asymptotic Correlation Matrix of Parameter Estimates

( *** The model parameter(s) -slope

have been estimated at a boundary point, or have been specified by the user,

and do not appear in the correlation matrix )

background intercept

background 1 -0.45

intercept -0.45 1

Parameter Estimates

95.0% Wald Confidence Interval

Variable Estimate Std. Err. Lower Conf. Limit Upper Conf. Limit

background 0.333332 * * *

intercept -4.01656 * * *

slope 18 * * *

* - Indicates that this value is not calculated.

Analysis of Deviance Table

Model Log(likelihood) # Param's Deviance Test d.f. P-value

Full model -15.2763 3

Fitted model -15.2764 2 6.10343e-005 1 0.9938

Reduced model -22.9145 1 15.2763 2 0.0004817

AIC: 34.5527

Goodness of Fit

Scaled

Dose Est._Prob. Expected Observed Size Residual

------------------------------------------------------------------------

0.0000 0.3333 4.000 4.000 12 0.000

1.2500 0.6667 8.000 8.000 12 -0.000

2.5000 1.0000 12.000 12.000 12 0.006

Chi^2 = 0.00 d.f. = 1 P-value = 0.9956

Benchmark Dose Computation

Specified effect = 0.1

Risk Type = Extra risk

Confidence level = 0.95

BMD = 1.10636

BMDL = 0.273501

Endpoint 2: Focal necrosis

====================================================================

Logistic Model. (Version: 2.14; Date: 2/28/2013)

Input Data File: C:/USEPA/BMDS250/Data/lnl_Nicotine Yuen et al. 1995_Nicotine Yuen et al. 1995.(d)

Gnuplot Plotting File: C:/USEPA/BMDS250/Data/lnl_Nicotine Yuen et al. 1995_Nicotine Yuen et al. 1995.plt

Thu Feb 05 09:55:31 2015

====================================================================

BMDS_Model_Run

~~~~~~~~~~~~~~~~~~~~~~~~~~~~~~~~~~~~~~~~~~~~~~~~~~~~~~~~~~~~~~~~~~~~~

The form of the probability function is:

P[response] = background+(1-background)/[1+EXP(-intercept-slope*Log(dose))]

Dependent variable = FocalNecrosis

Independent variable = Dose

Slope parameter is restricted as slope >= 1

Total number of observations = 3

Total number of records with missing values = 0

Maximum number of iterations = 500

Relative Function Convergence has been set to: 1e-008

Parameter Convergence has been set to: 1e-008

User has chosen the log transformed model

Default Initial Parameter Values

background = 0.25

intercept = -0.741267

slope = 4.32193

Asymptotic Correlation Matrix of Parameter Estimates

( *** The model parameter(s) -slope

have been estimated at a boundary point, or have been specified by the user,

and do not appear in the correlation matrix )

background intercept

background 1 -0.38

intercept -0.38 1

Parameter Estimates

95.0% Wald Confidence Interval

Variable Estimate Std. Err. Lower Conf. Limit Upper Conf. Limit

background 0.25 * * *

intercept -3.79342 * * *

slope 18 * * *

* - Indicates that this value is not calculated.

Analysis of Deviance Table

Model Log(likelihood) # Param's Deviance Test d.f. P-value

Full model -14.3862 3

Fitted model -14.3862 2 5.49311e-005 1 0.9941

Reduced model -23.546 1 18.3196 2 0.0001052

AIC: 32.7724

Goodness of Fit

Scaled

Dose Est._Prob. Expected Observed Size Residual

------------------------------------------------------------------------

0.0000 0.2500 3.000 3.000 12 0.000

1.2500 0.6667 8.000 8.000 12 -0.000

2.5000 1.0000 12.000 12.000 12 0.005

Chi^2 = 0.00 d.f. = 1 P-value = 0.9958

Benchmark Dose Computation

Specified effect = 0.1

Risk Type = Extra risk

Confidence level = 0.95

BMD = 1.09273

BMDL = 0.241143

Endpoint 3: Dark cell change

====================================================================

Multistage Model. (Version: 3.4; Date: 05/02/2014)

Input Data File: C:/USEPA/BMDS250/Data/msc_Nicotine Yuen et al. 1995_Nicotine Yuen et al. 1995.(d)

Gnuplot Plotting File: C:/USEPA/BMDS250/Data/msc_Nicotine Yuen et al. 1995_Nicotine Yuen et al. 1995.plt

Thu Feb 05 10:05:08 2015

====================================================================

BMDS_Model_Run

~~~~~~~~~~~~~~~~~~~~~~~~~~~~~~~~~~~~~~~~~~~~~~~~~~~~~~~~~~~~~~~~~~~~~

The form of the probability function is:

P[response] = background + (1-background)*[1-EXP(

-beta1*dose^1-beta2*dose^2)]

The parameter betas are restricted to be positive

Dependent variable = Darkcellchange

Independent variable = Dose

Total number of observations = 3

Total number of records with missing values = 0

Total number of parameters in model = 3

Total number of specified parameters = 0

Degree of polynomial = 2

Maximum number of iterations = 500

Relative Function Convergence has been set to: 1e-008

Parameter Convergence has been set to: 1e-008

Default Initial Parameter Values

Background = 0.084713

Beta(1) = 0

Beta(2) = 0.3728

Asymptotic Correlation Matrix of Parameter Estimates

( *** The model parameter(s) -Beta(1)

have been estimated at a boundary point, or have been specified by the user,

and do not appear in the correlation matrix )

Background Beta(2)

Background 1 -0.47

Beta(2) -0.47 1

Parameter Estimates

95.0% Wald Confidence Interval

Variable Estimate Std. Err. Lower Conf. Limit Upper Conf. Limit

Background 0.203839 * * *

Beta(1) 0 * * *

Beta(2) 0.26957 * * *

* - Indicates that this value is not calculated.

Analysis of Deviance Table

Model Log(likelihood) # Param's Deviance Test d.f. P-value

Full model -17.8282 3

Fitted model -18.6431 2 1.62984 1 0.2017

Reduced model -24.9533 1 14.2501 2 0.0008047

AIC: 41.2863

Goodness of Fit

Scaled

Dose Est._Prob. Expected Observed Size Residual

------------------------------------------------------------------------

0.0000 0.2038 2.446 3.000 12.000 0.397

1.2500 0.4775 5.730 4.000 12.000 -1.000

2.5000 0.8523 10.228 11.000 12.000 0.628

Chi^2 = 1.55 d.f. = 1 P-value = 0.2128

Benchmark Dose Computation

Specified effect = 0.1

Risk Type = Extra risk

Confidence level = 0.95

BMD = 0.625177

BMDL = 0.213503

BMDU = 0.908647

Taken together, (0.213503, 0.908647) is a 90 % two-sided confidence

interval for the BMD

Cancer Slope Factor = 0.468378
